# Bright Electrically Contacted Circular Bragg Grating Resonators with Deterministically Integrated Quantum Dots

**DOI:** 10.1021/acsnano.4c07820

**Published:** 2024-11-05

**Authors:** Setthanat Wijitpatima, Normen Auler, Priyabrata Mudi, Timon Funk, Avijit Barua, Binamra Shrestha, Johannes Schall, Imad Limame, Sven Rodt, Dirk Reuter, Stephan Reitzenstein

**Affiliations:** † Institute of Solid State Physics, Technische Universität Berlin, Hardenbergstraße 36, Berlin 10623, Germany; ‡ Department of Physics, Paderborn University, Warburger Str. 100, 33098 Paderborn, Germany

**Keywords:** semiconductor quantum dots, single-photon sources, circular Bragg grating, electro-optics, photon extraction efficiency, deterministic integration

## Abstract

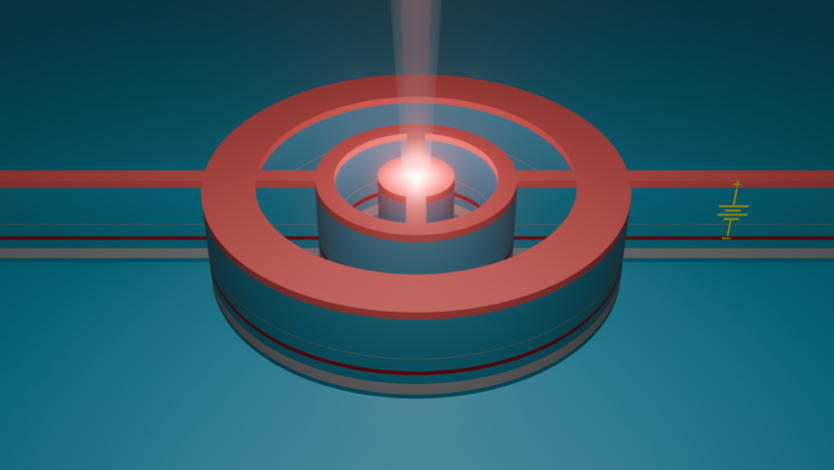

Cavity-enhanced emission of electrically controlled semiconductor quantum dots (QDs) is essential in the development of bright quantum devices for real-world quantum photonic applications. Combining the circular Bragg grating (CBG) approach with a PIN-diode structure, we propose and implement designs for ridge-based electrically contacted QD-CBG resonators. Through fine-tuning of device parameters in numerical simulations and deterministic nanoprocessing, we produced electrically controlled single QD-CBG resonators with excellent electro-optical emission properties. These include multiple wavelength-tunable emission lines and a photon extraction efficiency (PEE) of up to 30.4(3.4)%, where refined numerical optimization based on experimental findings suggests a substantial improvement, promising PEE > 50%. Additionally, the developed quantum light sources yield single-photon purity reaching 99.2(2)% and photon indistinguishability of 75(5)% under quasi-resonant p-shell excitation. Our results present high-performance quantum devices with combined cavity enhancement and deterministic charge-environment controls, which are relevant for the development of photonic quantum information systems such as complex quantum repeater networks.

## Introduction

In the field of photonic quantum information processing, the concept of quantum repeaters^1^ has emerged as a cornerstone of advanced quantum communication, serving as a key element to extend the communication range limited in simple point-to-point concepts by absorption to distances below about 100 km and thereby enabling high bit-rate long-range transmission.^2^ Implementing quantum repeater networks requires on-demand single-photon sources with high single-photon purity and indistinguishability as well as wavelength tunability and electrically controllable spin-photon interfaces.^3,4^ These stringent criteria have driven extensive research efforts, encompassing the exploration of suitable material systems and tailoring practical device designs for real-world applications.^5^ In this context, semiconductor quantum dots (QDs) have been the most outstanding candidates for single-photon sources in quantum information technology scenarios.^6^ Not only do they exhibit excellent quantum optical properties, but based on semiconductor materials, they also allow for device integration using advanced deterministic nanofabrication techniques to enhance their optical performance.^7^

For applications in photonic quantum information technology, external control over the electronic states of QDs is of great importance, for instance, to bring QDs in remote quantum light sources into spectral resonance which is needed to enable entanglement distribution via Bell-state measurements in quantum repeater networks.^8,9^ In this aspect, researchers have reported results on electrical charge control of QDs embedded within field-effect structures such as PIN diodes,^10−13^ and applied quantum-confined Stark effect^14^ for spectral fine-tuning.^15−17^

Parallel studies have focused on enhancing the photon extraction efficiency (PEE) of QD quantum light sources, for instance, via nanophotonic cavities. Among these cavities, circular Bragg grating (CBG) resonators have gained prominence due to their broadband emission enhancement in combination with pronounced light-matter interaction in the Purcell regime of cavity quantum electrodynamics.^4,18−20^ A CBG resonator is typically fabricated by etching a series of circular trenches around a central disk with the embedded targeted QD, creating a high refractive index contrast to realize tight lateral confinement of the light field.^21,22^ When a CBG resonator is paired with a back-side mirror, such as gold or a distributed Bragg reflector (DBR), redirecting the emitted photons upward, the vertical collection efficiency can be significantly boosted to PEE values exceeding 80% in experiments.^4,20^ However, due to the described geometry of a CBG resonator, a QD integrated inside such a structure is fundamentally electrically isolated. As a consequence, while the CBG-QD devices have performed well as on-demand devices under optical pumping,^4,20^ the experimental demonstration of electrically contacted CBG resonators has remained elusive.^23^ Notably, electrically contacted QD molecule devices with a PIN-diode design and surface CBG resonators were demonstrated, achieving experimental PEEs up to 24%, limited by only partial utilization of the CBG concept.^13^

**Figure 1:**
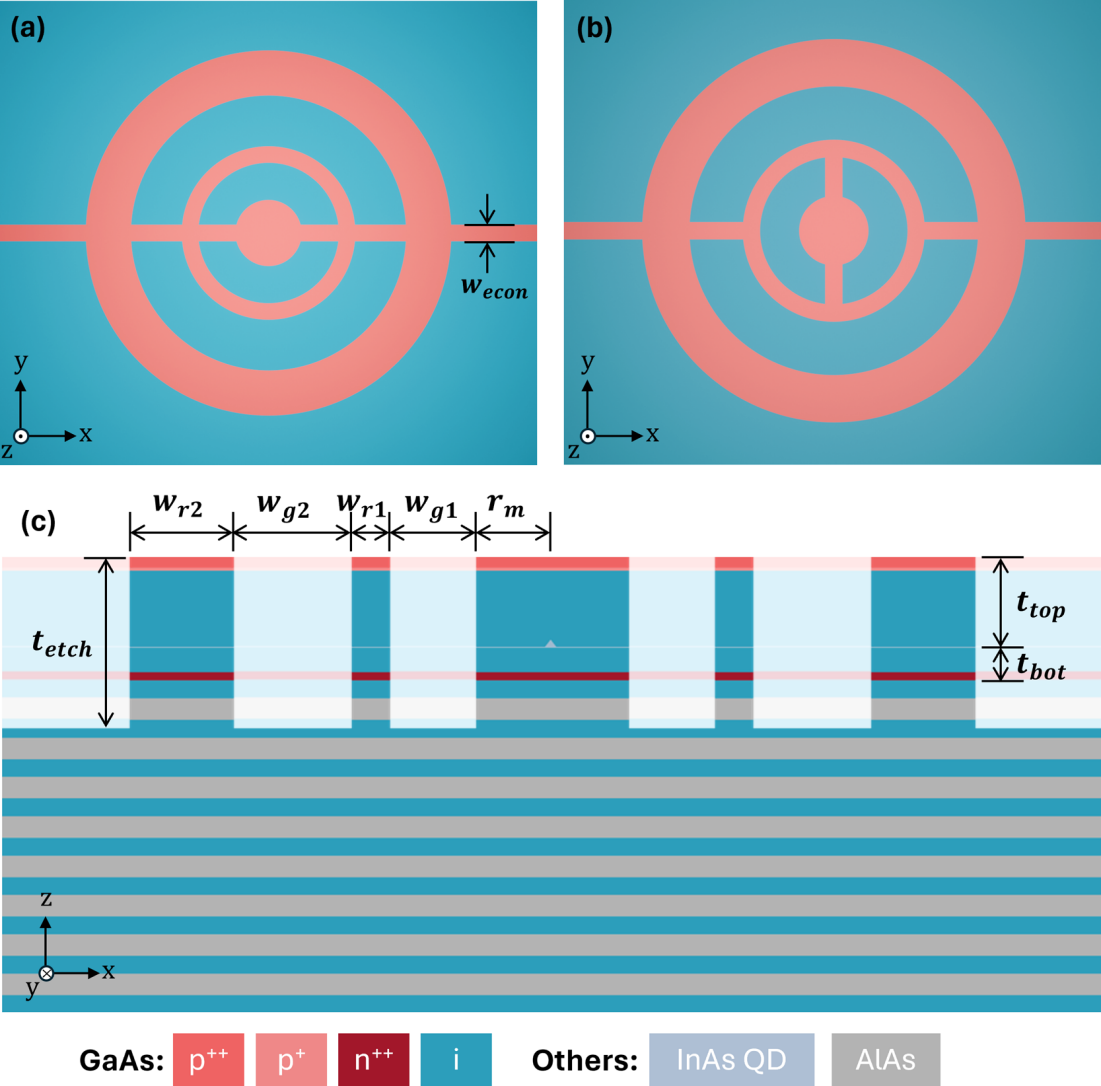
Design schematics of an electrically tunable single-photon source based on a QD integrated into an eCBG in (a) direct-ridge and (b) mazy-ridge configurations. (c) Vertical cross-section of the device illustrates a QD embedded in a PIN diode structure with back-side DBR consisting of 10 λ/4 thick mirror pairs. The device parameters denoted in the schematics include central mesa radius (*r*_m_), ring/gap widths (*w*_r1,r2/g1,g2_), ridge width (*w*_econ_), etch depth (*t*_etch_), and QD top/bottom spacer (*t*_top/bot_).

To solve the issue of implementing high-brightness CBG single-photon sources with electrical control of integrated QDs, recent design efforts have explored ridge-based CBG approaches.^24−27^ In these methods, instead of etching complete circular trenches around QDs as done on optically pumped structures, narrow ridges are unetched to retain the connection of doped layers in the central mesa to the area outside of the resonator where metallic pads can be flexibly prepared for external electrical control (see schematic drawings in Figure 1). However, to date, most works have only reported numerical designs of such modified CBGs and discussed theoretical aspects of the optical performance.^23,25,27,28^ Only one experimental work has been reported, where CBG structures with tapered bridges were applied, aiming to stabilize the charge environment of QDs; however, no electro-optical effects have been shown to verify electrical control of QD properties by the introduced bridges,^26^ in contrast to the earlier works done on electrically controlled planar QDs and micropillars.^15−17^ The relevant CBG works are summarized in Supporting Information, Table S1.

Therefore, we provide experimental results on electrically controlled QDs in CBG resonators. We first discuss aspects of designing an electrically contacted CBG (eCBG) with an integrated QD along with numerical simulations to maximize its PEE, and then discuss the device fabrication and the optical characterization. The design utilizes a DBR as the back-side mirror to simplify the device fabrication process, instead of using a gold mirror, which requires a postgrowth flip-chip process.^4,20^ In this scenario, the heterostructure, including DBR, QDs, and the PIN diode, can be grown epitaxially in a single process using metal–organic chemical vapor deposition or molecular beam epitaxy (MBE). The deterministic nanofabrication process utilizes marker-based electron-beam lithography (EBL) in combination with low-temperature cathodoluminescence (CL) mapping to determine the positions and spectral features of suitable QDs before device integration. To investigate the electro-optical performance of the fabricated eCBG devices and assess the quantum optical properties of the deterministically integrated QDs, bias-voltage dependent microphotoluminescence (μPL) measurements and time-resolved photon correlation measurements are performed.

## Results and Discussion

### Device Design

Designing a nanophotonic device involves careful consideration of the interplay between the geometry of individual structural elements in the proximity of the photon source (in this case, a QD) and their respective refractive indices. When modifying the well-known optically pumped CBG resonator concept into a ridge-based eCBG, the introduction of contact ridges which can lead to scattering and lateral photon losses is the primary factor influencing, and potentially reducing, the PEE. In this context, two aspects related to the electrical contacts are considered in particular: the electrical contact path length and the width of the ridge.

From an electrical perspective, the ridge should ideally have the shortest path length connecting the central mesa to the external areas to minimize the electrical resistance. This condition constitutes our first proposed design featuring direct-ridge electrical connections, illustrated in Figure 1a. In this design, when considering the optical perspective, such a straight ridge could provide an undesired optical loss channel for emitted photons from the central mesa via waveguiding, thereby deteriorating the lateral confinement. In fact, as a countermeasure, labyrinth-like structures have been proposed, where the connections between rings were sectioned and rotated.^27^ As a comparative experimental case study, another design featuring mazy-ridge electrical connections to reduce the effect of lateral waveguides is also proposed here and is depicted in Figure 1b. To keep the electrical path as short as possible, both designs feature only two rings, and in the mazy-ridge design, only the connections inside the first ring are rotated.

A similar trade-off scenario applies to the width of the ridge as well. For electrical purposes, the ridge width should ideally be as large as possible to minimize the resistance, but as the ridge becomes wider, it could support the propagation of waveguiding modes,^29,30^ degrading the optical performance of the device. While the optical performance could be predicted and optimized via numerical simulations, the sensible prediction for the electrical behavior of nanoscale structures requires experimental insights, for instance, on the width of depleted layers at the etched surfaces. In fact, our eCBGs are patterned and fabricated utilizing inductively coupled plasma reactive-ion etching (ICP-RIE), which usually results in residual surface defects with deteriorated electrical conductivity.^31^ As a consequence, a functional ridge must be wide enough to accommodate an electrically active region sided by defective surfaces. For this, the minimum ridge width was initially selected as 100 nm, and the maximum ridge width was intuitively limited by the effective wavelength of typical InGaAs QD emissions (>900 nm) in the GaAs medium, which is about 250 nm.

Based on the considerations mentioned above, numerical optimizations were performed to identify the optimal device parameters depicted in Figure 1c by applying a Bayesian optimization algorithm to maximize the PEE at the numerical aperture (NA) of 0.81, which is the NA of the optics later used in the experiments. The field distributions and PEE were calculated by a three-dimensional (3D) finite-element method (FEM) in JCMsuite^32^ based on the direct-ridge configuration, exploiting two vertical mirror planes (one along the main ridge axis and one perpendicular to it) to reduce the demanding computational time and resources for full 3D FEM simulations. To sensibly limit the computation time for optimizations, the overall size of the simulated structures was bound by setting search ranges for the mesa radius (*r*_m_), ring widths (*w*_r1,2_), gap widths (*w*_g1,2_), and top/bottom-spacer (*t*_top/bot_) within 100 and 500 nm. The QD was modeled using a linearly polarized point-like dipole oriented 45° to the *x*-axis emitting at the wavelength of 930 nm, and the absorption effect of dopants in p- and n-doped GaAs was neglected in the simulation at this wavelength.

The device parameters obtained from the numerical optimization and the calculated PEEs for both designs are given in Table 1. In this device design, the top spacer (*t*_top_) and bottom spacer (*t*_bot_), which distance the QDs from the surface and the DBR, were determined to be 333 and 119 nm, respectively. The uppermost part of the top spacer was chosen as a 50 nm p-doped layer, while the lowermost part of the bottom spacer was chosen as a 30 nm n-doped layer. It is important to point out that the optimal *w*_econ_ value for the highest PEE was found at the lower boundary of the defined search domain, which was 100 nm, implying that a narrow ridge width was favored. This numerical study, which predicts PEEs of 43.2 and 42.1% for the two considered eCBG configurations, confirmed the assumptions mentioned earlier regarding the ridge width and implied that the main limiting criterion for a functional ridge width is the electrical conductivity which needed to be investigated experimentally. The simulated effects of QD-eCBG spatial mismatch based on the two configurations are shown in Supporting Information, Figure S1.

**Table 1:** Optimized Device Parameters and Simulated PEEs Obtained from the Optimizations with the Minimum *w*_econ_ of 100 nm and Other Parameter Search Ranges Limited to the Maximum of 500 nm (Except for *t*_etch_, Which Was Limited to the Maximum of 800 nm)^a^

**structure**	**device parameters (nm)**					**sim. PEE (%)**
	*w*_econ_	***r*_m_**	*w*_g1,2_	*w*_r1,2_	*t*_etch_	
direct-ridge	100	243	268, 369	120, 327	630	43.2
mazy-ridge	100	243	268, 369	120, 327	630	42.1

a The PEEs (NA = 0.81) were calculated using a linearly polarized dipole oriented 45° to the *x*-axis emitting at the wavelength of 930 nm.

### Deterministic Fabrication

Following the aforementioned optimized design parameters, the heterostructure was epitaxially grown using MBE as follows: First, at a substrate temperature of 605 °C, a 100 nm GaAs buffer layer was deposited on an undoped GaAs (100) wafer, followed by the growth of a DBR consisting of ten pairs of AlAs and GaAs layers with nominal thicknesses of 79 and 67 nm, respectively. Subsequently, a 30 nm Si-doped GaAs layer with a doping concentration of 2 × 10^18^ cm^–3^ was grown at 555 °C. Then, the substrate temperature was returned to 605 °C, and an 86 nm GaAs layer was deposited. Afterward, a 3 min pause was introduced during which the substrate temperature was lowered from 605 to 505 °C, and the arsenic pressure was gradually reduced from 2.25 × 10^–5^ to 1.50 × 10^–5^ mbar. After a 2 min temperature stabilization period, 3 nm of GaAs were deposited. Indium was then deposited in a pulsed mode (4 s of deposition, 4 s of pause) over 20.5 deposition cycles. During the initial 10.5 cycles, the substrate rotation was halted to create an indium gradient along the [110] direction. The rotation was resumed for the remaining 10 cycles at a speed of 10 rpm. The QDs were then partially capped with 2.6 nm of GaAs at 485 °C. Following this, the temperature was rapidly increased to 605 °C, and a 280 nm GaAs layer was grown. Lastly, a top gate structure consisting of 10 and 40 nm C-doped GaAs layers, with doping concentrations of 1 × 10^18^ and 1 × 10^19^ cm^–3^, respectively, was deposited at 555 °C. All temperatures mentioned were determined using band-edge thermometry.

Following the epitaxial growth, a sample piece of (5 × 5) mm^2^ with low QD density (about 2.5 × 10^6^ cm^–2^) was selected, on which electrical contacts and Au markers were prepared via four EBL steps. The first lithography was performed to pattern areas at the sample corners, which were then etched by 410 nm using ICP-RIE to remove the top p-doped layer and prepare for n-pads. Then, within the etched areas, n-contact pads were patterned and deposited with thicknesses of 10 nm of Ni, 50 nm of Au_0.88_Ge_0.12_, and 40 nm of Au. After that, the sample was rapidly annealed at 420 °C for 90 s with a 500 K/min ramping rate under a N_2_ atmosphere. For the third EBL, p-contacts and markers were patterned, and 10 nm Ti and 90 nm Au were deposited. Lastly, all p- and n-contacts were thickened with an additional 250 nm Au to achieve the total pad thickness of 350 nm to ensure the durability of the pads during the wire bonding procedure.

**Figure 2:**
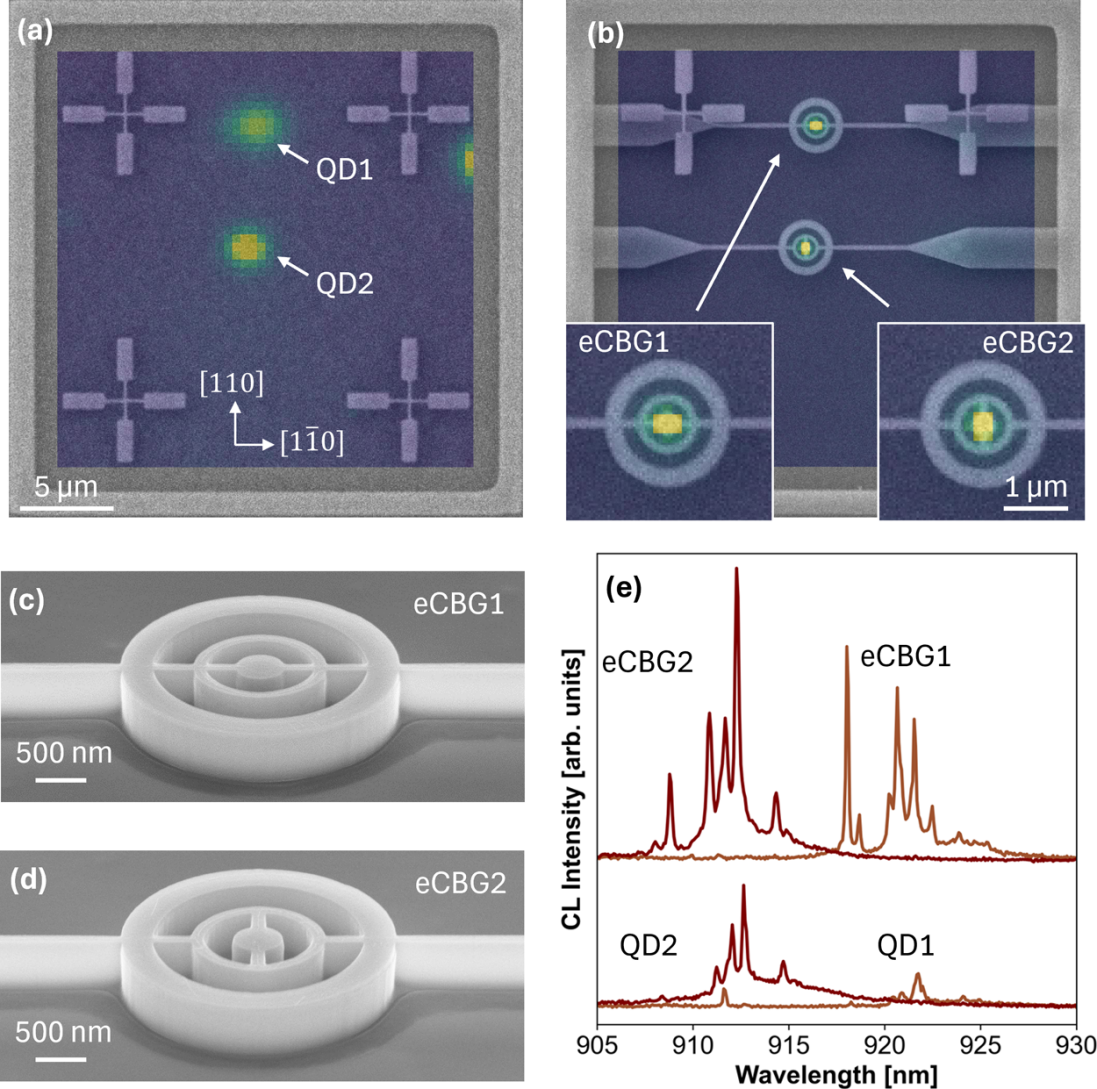
(a) CL map overlaid on the SEM picture of a marker field, showing two spatially localized QDs (labeled QD1 and QD2) in a planar structure. (b) CL map of the same marker field after using marker-based EBL to pattern eCBGs on the QDs deterministically with the main ridge axis aligned along the [11̅0] orientation. QD1 was integrated into a direct-ridge eCBG, whereas QD2 was integrated into a mazy-ridge eCBG. (c,d) SEM images of the fully processed eCBG structures. (e) CL spectra of QD1 and QD2 before (bottom) and after (top) the integration into eCBGs.

Suitable QDs were identified based on their emission wavelength and separation from other QDs on the sample with prepatterned markers by scanning each marker field using CL mapping at 20 K without applying any bias voltage (open-circuit). A map consisting of an array of CL spectra and a scanning electron microscopy (SEM) image, as illustrated using an exemplary field in Figure 2a, provides spectral and spatial information about suitable QDs, which can be preselected for device fabrication. After identifying the QDs, marker-based EBL^33^ and ICP-RIE were performed to pattern eCBGs at the determined QD positions. As the definitive minimum ridge width for an electrically active eCBG was unknown, the ridge width was intentionally varied from 100 to 130 nm on different patterned structures without changing any other parameters.

The enhancement from the eCBG integration on the QDs could be observed via a one-to-one comparison by performing another CL mapping on the same marker field after the integration, as depicted in Figure 2b, showing that devices were patterned on the determined QDs. Note that single QD emission spectra could be observed on all 14 deterministically fabricated eCBGs. Therefore, we conclude that the fabrication yield of the working devices is close to 100%. In the marker field, two QDs (namely QD1 and QD2) were integrated into a direct-ridge structure (eCBG1) and a mazy-ridge structure (eCBG2), whose SEM images are illustrated in Figure 2c,d, respectively. The images indicate smooth etched surfaces and well-defined fabrication conditions. Figure 2e shows the CL spectra of both QDs before and after device integration using the same excitation and collection parameters. It is important to note that the intensity between QDs could not be compared fairly as the CL mapping was performed using a parabolic mirror, which resulted in a nonuniform intensity profile throughout the map. Qualitatively, the postintegration spectra of both QDs clearly indicate strongly enhanced extraction efficiency and improved signal-to-noise ratio.

### Electric-Field-Dependent Photoluminescence Measurements

**Figure 3:**
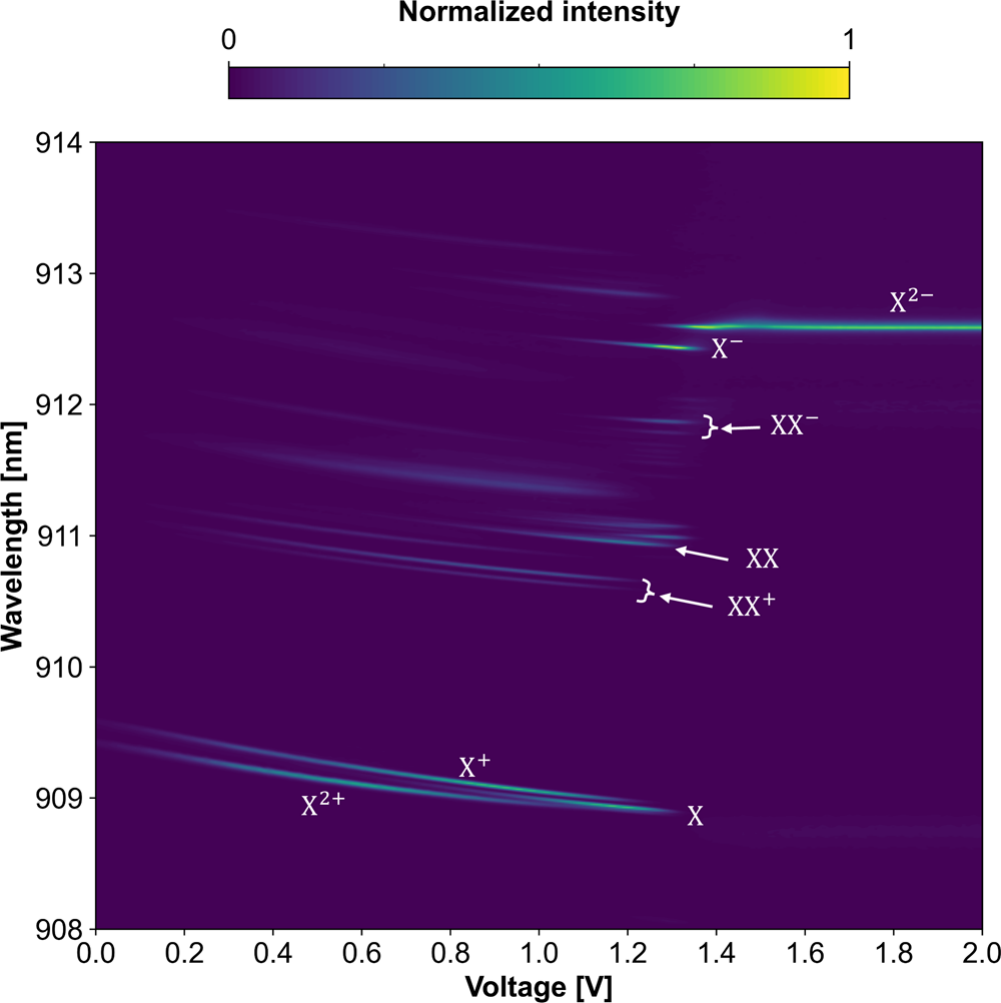
Contour plot of bias-voltage dependent μPL spectra of the eCBG2-QD recorded at 4 K showing several emission lines originating from neutral excitonic (X), biexcitonic (XX), singly charged (X^±^, XX^±^), and doubly charged (X^2+/2–^) states observed at different bias voltages. The lines observed at bias voltages under 1.4 V exhibit spectral shifts due to the quantum-confined Stark effect, allowing for emission wavelength tuning via external electrical controls.

After the deterministic fabrication, electrical connections between an external voltage source and the diode structures of the fabricated devices were made through wire bonding between a chip carrier and metallic contact pads. The chip carrier was then installed in the optical setup described in Section 10 for bias-voltage dependent μPL measurements. The eCBGs were investigated under pulsed (80 MHz) picosecond-mode excitation via a wetting layer at 865 nm using a Ti:Sapphire laser. By varying the external bias voltage (*V*_ex_) between 0 and 2 V, we observed that the fabricated direct-ridge eCBGs whose ridges were narrower than 110(5) nm exhibited no voltage dependency on their optical spectra, as well as the mazy-ridge eCBGs whose ridges were narrower than 120(5) nm. Both eCBG1 and eCBG2, as well as other eCBGs that consisted of wider ridges than these values, showed clear and similar bias-voltage dependency, as illustrated using eCBG2 as an example in Figure 3.

The μPL contour plot displays different QD emission lines observed at different *V*_*ex*_. Most lines, which appeared below about 1.4 V, featured the quantum-confined Stark effect, by which the emission wavelengths shifted gradually over the *V*_ex_ change, whereas the emission lines appearing above 1.4 V remained energetically constant. This behavior can be explained by the electronic band-bending of the PIN diode structure, which was initially caused by the built-in electric field (*E*_in_).^14^ At zero bias, the electronic bands were strongly bent by the field which promotes the quantum tunneling rate of electrons from the wetting layer to GaAs, reducing carrier capturing into the QD; hence, the low PL emission rates were observed at 0 V.^34,35^ As the *V*_ex_ increased, the band-bending and electron tunneling weakened, enabling the carrier capturing in the QD and subsequent carrier recombination; hence, the emission lines became visible while shifting energetically.^15,35^ Notably, bias-induced energetic shifts of up to 0.7 meV were observed in the studied devices. When the externally applied electric field became as equally strong as the *E*_in_, which was around *V*_ex_ = 1.4 V), flat-band conditions were achieved, and no quantum confined Stark tuning could be observed anymore.

**Figure 4:**
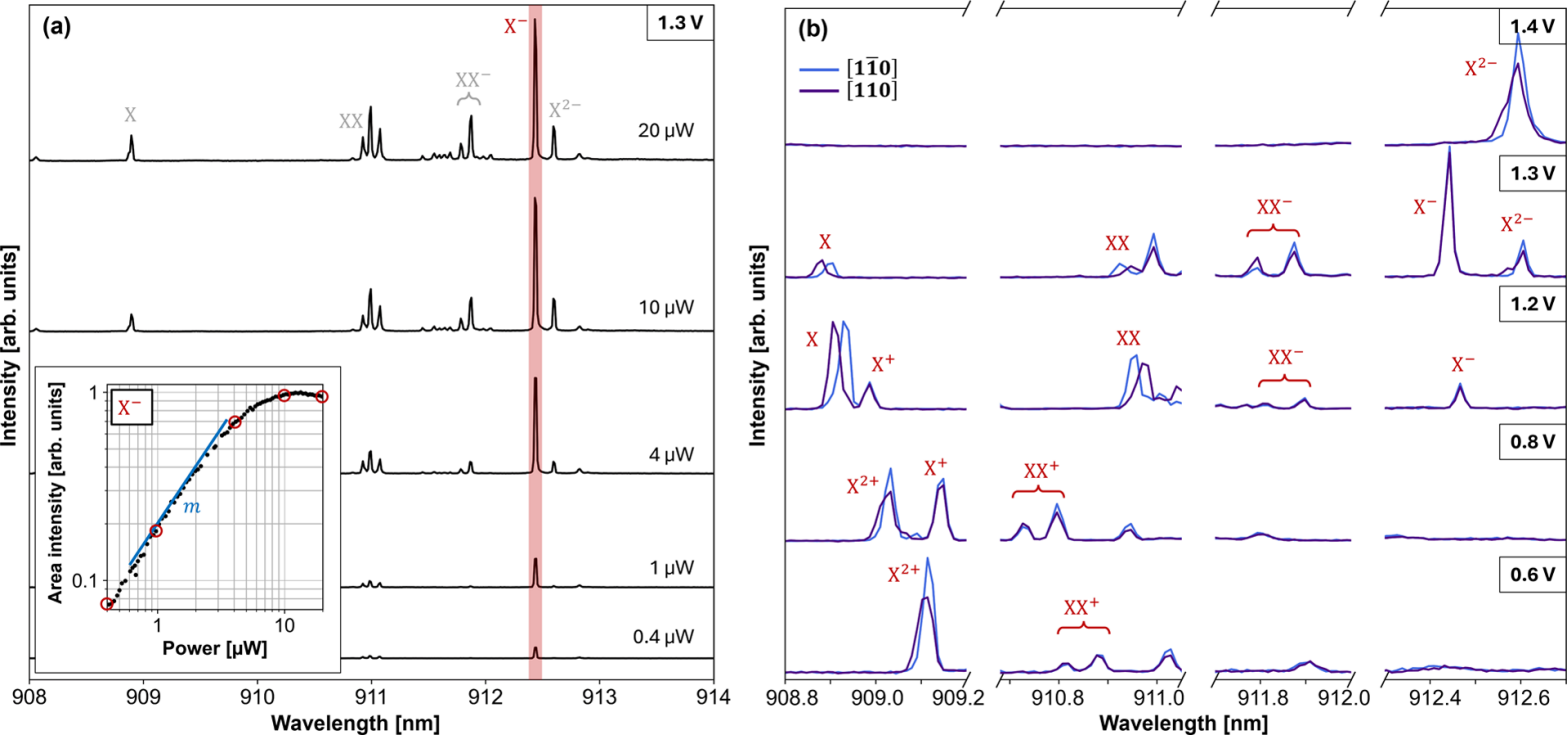
(a) Power-dependent μPL spectra from eCGB2 biased at 1.3 V, and the area intensity of the X^–^ emission line plotted against excitation power in a log–log scale. (b) Polarization-dependent μPL spectra from eCBG2 biased at different bias voltages, showing multiple emission lines. The two perpendicular polarization angles plotted correspond to the [11̅0] (blue) and [110] (purple) orientations of the sample.

The nature of the observed emission lines was determined by performing power- and polarization-dependent μPL measurements at the corresponding *V*_ex_. For the power-dependent measurements, the μPL spectra were recorded at different optical excitation powers. By selectively fitting the area intensity of each emission line and plotting it against the excitation power in a log–log scale, a power exponent (*m*) can be extracted from the slope of the linear section in the plot. Figure 4a shows an example of a power-dependent μPL measurement at 1.3 V where an emission line (later determined as the X^–^ emission line) was selectively fitted. The same process was done for other lines, and the findings are listed in Table 2. With extracted *m*’s, excitonic and biexcitonic lines were categorized by their linear (*m* ∼ 1) and superlinear (*m* > 1) power dependencies, respectively.^36^ Complementarily, the information on polarization properties of these emission lines was necessary to determine their origins unambiguously, caused by the spin-related fine structures.^37−39^ For this, the polarization-dependent measurements were performed by inserting a rotatable λ/2-plate and a linear polarizer in the collection path of the μPL setup. By change of the λ/2-plate angle, different polarization components of each emission line were recorded. Figure 4b shows the two polarization components of each emission line along [11̅0] and [110] orientations of the sample. For each line, the peak energy difference between the two polarization angles and the line width (full-width half-maximum measured at a certain polarization angle where the narrowest emission was observed) were determined and are also listed in Table 2. Combining the findings from these two measurements, the emission lines were determined as follows:

**Table 2:** Emission Lines Observed from eCBG2 at Different Bias Voltages and Their Relevant Parameters, Including Emission Wavelengths (λ) and Central Energies (*E*), Power Exponents (*m*) from Power-Dependent μPL Measurements, Fitted Peak Energy Deviations (Δ*E*) and Minimum Emission Line Widths (FWHM) from Polarization-Dependent μPL Measurements

emission	voltage (V)	λ (nm)	*E* (meV)	*m*	Δ*E* (μeV)	fwhm (μeV)
X^2+^	0.6	909.11	1366.58	1.51(1)	9.0(1.8)	31.5(2.9)
X^+^	0.8	909.14	1366.53	0.85(1)	none	23.7(2.5)
XX_1_^+^	0.8	910.73	1364.15	1.50(2)	none	24.8(4.6)
XX_2_^+^	0.8	910.80	1364.05	1.60(3)	none	29.7(3.1)
X	1.2	908.92	1366.87	0.82(1)	37.2(2.6)	24.6(4.7)
XX	1.2	910.96	1363.81	1.28(1)	34.4(2.4)	27.2(2.4)
XX_1_^–^	1.3	911.79	1362.57	1.69(4)	none	28.9(3.1)
XX_2_^–^	1.3	911.87	1362.44	1.78(2)	none	29.4(1.2)
X^–^	1.3	912.44	1361.60	0.97(1)	none	16.6(1.1)
X^2–^	1.4	912.59	1361.37	1.29(1)	7.7(1.4)	46.0(1.2)

First, the most fundamental states of QD, exciton-biexciton (X-XX) emissions, were observed at around 1.2 V, exhibiting a strong polarization dependency with the opposite energetic deviation direction. The energy deviations indicate the X-XX fine-structure splittings of 37.2(2.6) μeV for X and 34.4(2.4) μeV for XX, respectively, which are typical values for an InGaAs QD.^40^ Second, when varying *V*_ex_ slightly to a lower value (0.8 V), three prominent lines were observed without any polarization-dependent energetic variation, consisting of a single line with *m* ∼ 1 and double lines with *m* > 1. This suggests that they originate from singly charged excitonic and biexcitonic states. Moreover, the double lines exhibited clear polarization-dependent intensities, where each line was more prominent than the other at different polarization angles. This strongly supports that they originate from the relaxation of the charged biexcitonic state into the two triplet states of excited-trion, which typically generate elliptically polarized photons.^37,41^ Third, when decreasing *V*_ex_ even further (<0.8 V), a new prominent line appeared with *m* > 1 and showing a polarization-dependent emission. The emission was observed as a single line at a certain polarization angle, but the line broadened with a lower peak intensity when observed at different polarization angles. This suggests that the emission consists of two different lines, one without polarization dependency and another with polarization-dependent energetic variation, which could be attributed to the two triplet transitions from doubly charged excitonic states.^38,42^

Another similar set of lines was observed as well when varying the voltage to the higher values (*V*_ex_ > 1.2 V), indicating that they originate from the analogous spin states with the opposite charge sign. Note that, at lower voltages (*V*_ex_ < 1.2 V) where the band-bending exists, the tunneling rate of electrons is more significantly promoted than that of holes due to the smaller effective mass, suggesting the hole is more effectively captured into the QD and the prominent emission lines are expected to originate from the positively charged states.^34,35^ On the other hand, at higher voltages (*V*_ex_ > 1.2 V), where the tunneling weakens, the electron capturing in the QD becomes effective, suggesting that the emission lines in this regime originate from negatively charged states instead. Additionally, the redshifts of the higher negatively charged emission lines also support the assumptions, in agreement with previously reported studies.^42−44^ Consequently, all of the prominent emission lines in the voltage-dependent μPL spectra were assigned, ranging from the positive doubly charged line (X^2+^) at low voltages to the negative doubly charged line (X^2–^) at high voltages, as shown in Figure 3.

Understanding the spin-related origins of the emission lines is crucial for application in, for instance, quantum repeater networks, which require well-defined light-matter interactions between the flying and stationary qubits.^6^ The results presented in this section demonstrate the determination of the origins of multiple QD emission lines and confirm the capability of the designed eCBG devices, allowing for deterministic control of electronic states of QDs in the cavity. This feature can significantly enhance the performance of spin-photon interfaces, crucial for mediating photon entanglements in quantum repeater networks.^45^ The emission wavelength tunability of the QD in the cavity enabled by this design can also benefit the applications with quantum memories.^46^ Moreover, the design can be directly applied to QD molecules, which have promising applications in photonic cluster-state generation.^13,47−49^

### Optical Enhancements

As mentioned above, the electrically functioning eCBGs were experimentally found to require wider ridges than the originally optimized value (100 nm). For a fair comparison between the simulation and experimental results, the actual geometry of the fabricated structures (eCBG1 and eCBG2) was measured using SEM, and the PEEs were resimulated based on the measured structural parameters, which are listed in Table 3. Note that the experimental PEE can deviate from the simulated value due to the possible mismatch of the QD position and the integrated eCBG,^50^ which could not be determined in this work, limited by the small diameter of the central mesa.

**Table 3:** Structural Parameters of Fabricated Device Obtained from SEM Imaging (with Measurement Errors ≤5 nm) and the Simulated and Measured PEEs (NA = 0.81)^a^

structure	**device parameters (nm)**					**PEE (%)**		λ **(nm)**
	*w*_econ_	***r*_m_**	*w*_g1,2_	*w*_r1,2_	*t*_etch_	simulated	measured	
planar					0	1.75–2.32	1.69(59)	900–930
eCBG1	122	240	250, 368	137, 324	633	24.53	12.6(1.4)	918.1
eCBG2	132	245	240, 368	137, 328	633	31.93	30.4(3.4)	912.7

a The simulations were performed using a linearly polarized dipole oriented 45° to the *x*-axis emitting at the corresponding wavelengths to the measured emissions. For planar QDs, the PEE was simulated for the wavelength range of 900–930 nm and measured from nine different QDs.

To obtain experimental PEEs, the μPL setup efficiency was measured using a continuous-wave laser at the same wavelength as the QD emission, yielding a value of 7.3(8)%. Then, nine different QDs in the planar structure and the studied devices (eCBG1 and eCBG2) were electrically biased at 1.5 V to achieve the flat-band conditions for maximum electric-field dependent PL emissions,^34^ and the emission spectrum from each QD was recorded under pulsed (80 MHz) excitation at 800 nm using a Ti:Sapphire laser with the corresponding saturation optical pump power. The PEE was calculated using the formula:
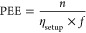
where *n* is the single-photon emission rate, η_setup_ is the setup efficiency, and *f* is the excitation frequency. For eCBG1 and eCBG2, single-photon emission rates were measured to be 732(18) kHz and 1.77(34) MHz, equivalent to PEEs of 12.6(1.4) and 30.4(3.4)%, respectively. For planar QDs, the average (and standard deviation) emission rate was found to be 98.6(26.1) kHz, which led to an average PEE of 1.69(59)%.

The comparison between the simulated and experimental PEE for eCBG2 shows an impressive conformity, indicating accurate numerical modeling and good fabrication quality. The reliability of this comparison can be supported by the results from planar QDs, which also show good agreement between the simulation and the measured value. Deviated from the simulation, eCBG1 yielded noticeably lower efficiency, similar to the other two fabricated direct-ridge eCBGs with the same nominal parameters. The cause for the larger deviation of the experimental PEE from the simulated one for eCBG1 than that of eCBG2 may be multifactored. First, although all eCBGs were fabricated under identical conditions since the actual eCBG-to-QD position offsets could not be determined, the effect of alignment mismatch cannot be completely ruled out. Second, the simulations were performed with a QD modeled as a linearly polarized point-like dipole oriented 45° to the ridge axis, which might not represent the actual QD emission well enough in the presence of ridge-like structures breaking the cylindrical symmetry. As a result, the simulation may have underestimated the undesired direct-ridge effect, causing a larger difference between the simulated and experimental PEEs only for direct-ridge eCBGs. An additional finding described as follows supports this assumption further.

**Figure 5:**
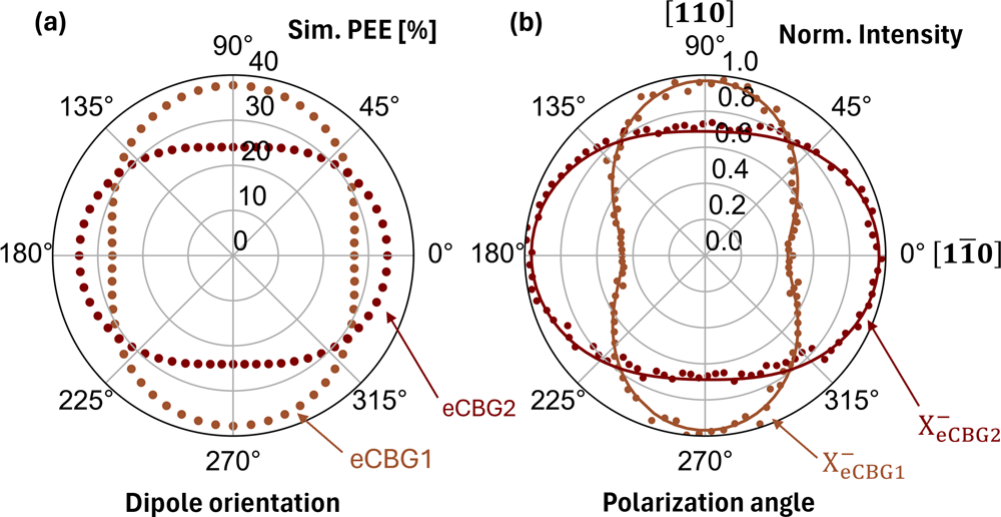
(a) Simulated PEEs (NA = 0.81) of eCBG1 and eCBG2 using a linearly polarized point-like dipole source oriented at different angles related to the main ridge axis. (b) Normalized intensities of the X^–^ emission lines from eCBG1 and eCBG2 in polarization-dependent PL measurements, where the 0° and 90° correspond to the linear polarization components along [110] and [11̅0] orientations, respectively.

Due to the presence of the ridges in eCBGs, the polarization-dependent optical performance of the fabricated devices was intuitively anticipated. On the simulation side, the effect was predicted by calculating the PEE using a linearly polarized dipole source oriented at different angles related to the main ridge axis. The simulation results for eCBG1 and eCBG2, plotted in Figure 5a, illustrate good enhancements for the dipoles oscillating perpendicularly to the innermost ridge axis, whereas the enhancements deteriorated the most when the dipole was placed in parallel instead. The simulated PEEs could be normalized and calculated into a degree of linear polarization: DLP = (*I*_max_ – *I*_min_)/(*I*_max_ + *I*_min_), where *I*_max/min_ is the maximum and minimum intensity (equivalent to PEE). Interestingly, both eCBG1 and eCBG2 yielded the same simulated DLP of 17%, regardless of the difference in the ridge continuity. On the experimental side, the effect could be observed in polarization-dependent μPL measurements mentioned in the previous section. To exemplify the effect, the X^–^ emission line (912.44 nm), which is intrinsically circularly polarized, was selected, and the fitted polarization-dependent intensity plots are shown in Figure 5b. Note that the polarization angle of 0° corresponded to the polarization components along the [11̅0] orientation of the sample to which the main ridge axes of the fabricated devices were aligned. Qualitatively, the experimental plots closely match the simulation results well, clearly confirming the polarization dependence of PEEs. The experimental DLPs were calculated in the same fashion as earlier, yielding 36(1) and 17(1)% for eCBG1 and eCBG2, respectively. For eCBG1, the experimental result implies poorer enhancement of the emission along the main ridge axis than the simulation predicted, which was another piece of evidence signifying the underestimation of the direct-ridge effect on the optical enhancement of the eCBG. On the other hand, another perfect match between simulation and experimental results on eCBG2 could be observed for DLP values. Thus, we conclude that this polarization-dependency of PEEs is mostly affected by the innermost ridges, which suggests that the outer ridges can be modified (e.g., for larger widths) to support electrical conductivity without significantly degrading the optical property.

To demonstrate the capability of improving the current designs, another numerical optimization was performed by setting *w*_econ_ to 120 nm and expanding the parameter search ranges for ring and gap widths to up to 1000 nm. The newly optimized parameters yielded the PEE > 50%, listed in Table 4, promising a substantial improvement without changing the heterostructure parameters. With numerical refinement, the proposed design can be adapted with more pairs of back-side DBR to boost the PEE even further.

**Table 4:** Optimized Device Parameters and Simulated PEEs Obtained from the Optimizations with the Minimum *w*_econ_ of 120 nm and Other Parameter Search Ranges up to a Maximum of 1000 nm^a^

**structure**	**device parameters (nm)**					**sim. PEE (%)**
	*w*_econ_	***r*_m_**	*w*_g1,2_	*w*_r1,2_	*t*_etch_	
direct-ridge	120	256	960, 607	257, 628	630	55.65
mazy-ridge	120	256	960, 607	257, 628	630	56.54

a The PEEs (NA = 0.81) were calculated using a linearly polarized dipole oriented 45° to the *x*-axis emitting at the wavelength of 930 nm.

### Single-Photon Characteristics

In addition to the optical performance of the fabricated devices, the quantum optical properties were also assessed by performing time-resolved μPL measurements and quantum optical measurements on the X^–^ emission line (912.44 nm) from eCBG2, as it appeared near the flat-band condition, resulting in a bright and spectrally narrow emission. The experiment was conducted under pulsed (80 MHz) picosecond-mode nonresonant and quasi-resonant excitation at 865 nm (wetting layer) and 899.5 nm (p-shell), respectively, using a Ti:sapphire laser in picosecond mode, and the detection path included a monochromator as a spectral filter and a photon correlation setup equipped with superconducting nanowire single-photon detectors (SNSPDs). For the nonresonant excitation measurements, the device, biased at 1.3 V, was optically pumped for 70% of the saturation intensity, while for the quasi-resonant excitation, the device, biased at 1.4 V, was operating at the saturation intensity.

**Figure 6:**
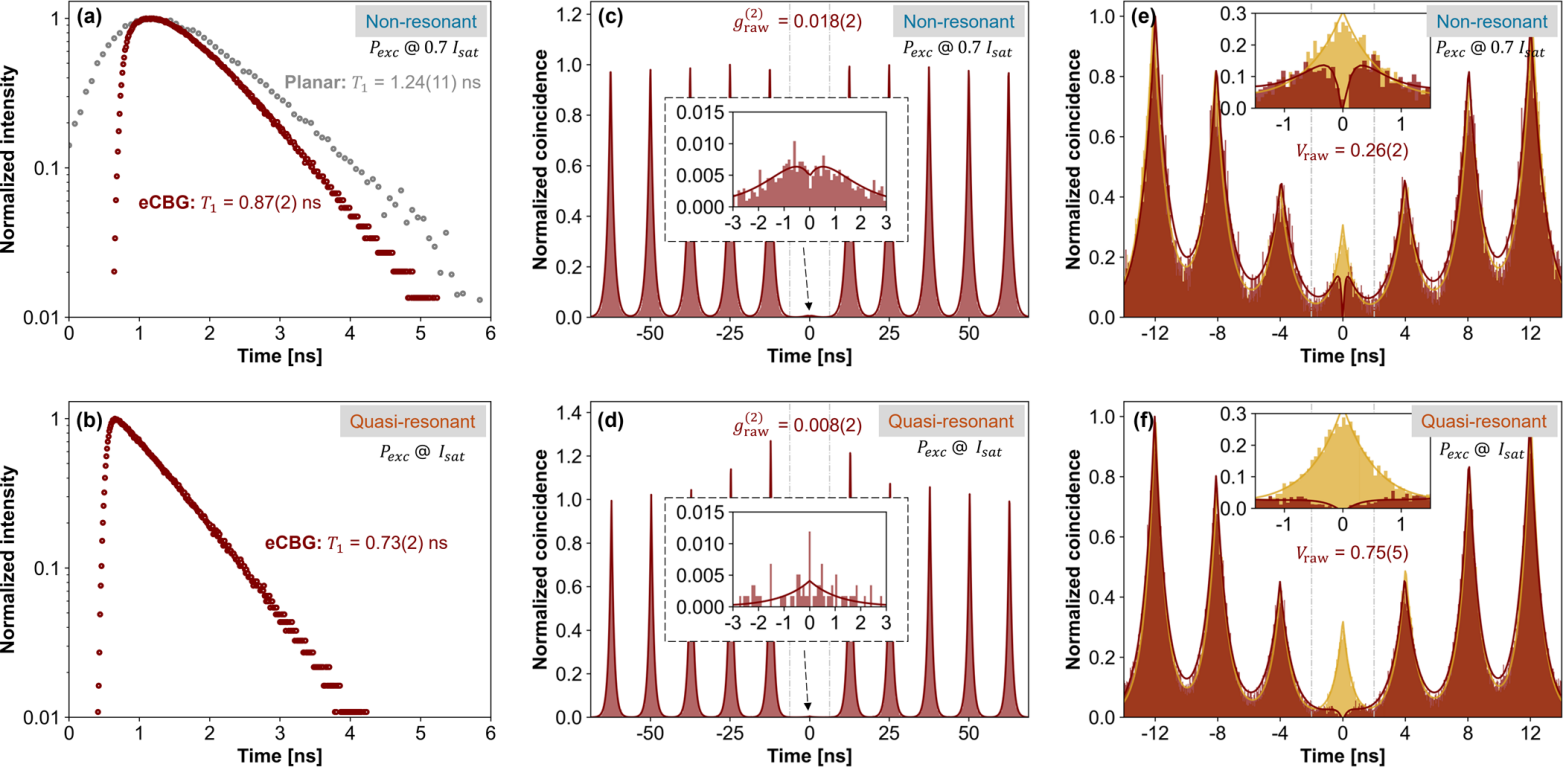
Time-resolved μPL measurements (a,b), measured Hanbury Brown and Twiss (c,d) and Hong-Ou-Mandel (e,f) autocorrelation functions of photons emitted from the X^–^ emission line of eCBG2 under nonresonant via wetting layer at 865 nm (top: a,c,e) and quasi-resonant via p-shell at 899.5 nm (bottom: b,d,f) excitation schemes. For non- (quasi-) resonant measurements, the device was optically pumped for 70% (100%) of the saturation intensity. In (a), an additional time-resolved measurement was performed on a planar QD in the same excitation scheme as a comparison. The *T*_1_ decay times were calculated by fitting the time-resolve measurements, while *g*_raw_^(2)^ values were obtained by comparing the middle peak coincidence (in a window of 12.5 ns between gray vertical dashed lines) and the mean of those from neighboring peaks. For the *V*_raw_ values, the middle peak coincidence (in a window of 4.2 ns) of the co- (maroon) and cross- (yellow) polarizations were compared.

At first, the device was investigated under a nonresonant excitation. The emission line width was determined by resolving the emission line through a scanning Fabry-Pérot interferometer (with a resolution limit of 150 MHz and a free spectral range of 12.3 GHz), yielding the full-width half-maximum (fwhm) of 1.31(2) GHz. Then, the time-resolved μPL measurements were performed to determine the *T*_1_ decay time of the device. From the results, illustrated in Figure 6a, the *T*_1_ decay time of the device was calculated as 0.87(2) ns, which is shorter than the decay time of a QD in the planar structure by 1.4(1) times, which can be considered a Purcell factor of the device. After that, a Hanbury Brown and Twiss (HBT) setup^51^ was used to assess the single-photon purity. The recorded photon autocorrelation histogram, plotted in Figure 6c, shows strong antibunching with a very low coincidence rate observed near the zero-time delay. The remaining coincidence counts near zero-time delay can be expected from carrier-recapturing processes due to the nonresonant excitation scheme. Nevertheless, the emission features an excellent multiphoton suppression rate with a *g*_raw_^(2)^ of 0.018(2), which is equivalent to the single-photon purities of 98.2(2)%. Finally, the photon indistinguishability was assessed using a Hong-Ou-Mandel (HOM) correlation setup.^52^ In this case, a sequence of double excitation pulses with a 4.2 ns pulse separation was used, which was matched with a 4.2 ns fiber delay line in one interferometer arm in the HOM setup. The HOM interference histograms from parallel and orthogonal polarization configurations are plotted in Figure 6e. The nonideal raw visibility was obtained with *V*_raw_ = 0.26(2), as evidenced by observed coincidences near the zero-time delay. These coincidences were consequences of the nonresonant excitation scheme, in which the carrier relaxed into the QD s-shell in an uncontrolled manner.^53^

After the nonresonant investigation, the same measurements were performed again under quasi-resonant p-shell excitation. Here, the emission line width was determined, having the fwhm of 1.01(3) GHz. As shown in Figure 6b, the *T*_1_ decay time of the device was shortened to 0.73(2) ns, resulting from the quicker carrier relaxation into the s-shell. Also, Figure 6d shows an almost perfect multiphoton suppression rate with a *g*_raw_^(2)^ of 0.008(2), leading to a single-photon purity of 99.2(2)%. This provides evidence of the vanishing of the recapturing process, as the excitation scheme generates only carriers in a discrete QD shell, which is bound by Pauli’s principle. Moreover, the generated photon indistinguishability was significantly improved with *V*_*raw*_ = 0.75(5).

One possibility to improve the photon indistinguishability even further is to perform measurements under strictly resonant excitation. However, in the case of CBG devices, whose mesa diameter can be as small as (or smaller than) the excitation laser beam diameter, the laser scattered by an etched profile can deteriorate the single-photon purity of the QD emission, limiting the visibility. In this case, the presence of ridges may enhance the scattering even further. As a countermeasure for such scenarios, eCBG, as well as CBG, devices may need to be optimized with an additional constraint for a larger mesa to circumvent the issue, as discussed above.

Another possibility is to exploit the confined cavity effect to reduce the radiative lifetime and minimize the spectral diffusion by optimizing the device for higher Purcell factors. It is noteworthy that although the current device exhibited only a weak Purcell enhancement, it still yields decent indistinguishability under a nonstrict resonant scheme. This indicates that the spectral diffusion over the rather long radiative lifetime was already significantly reduced by the well-defined charge environment under voltage control.

**Figure 7:**
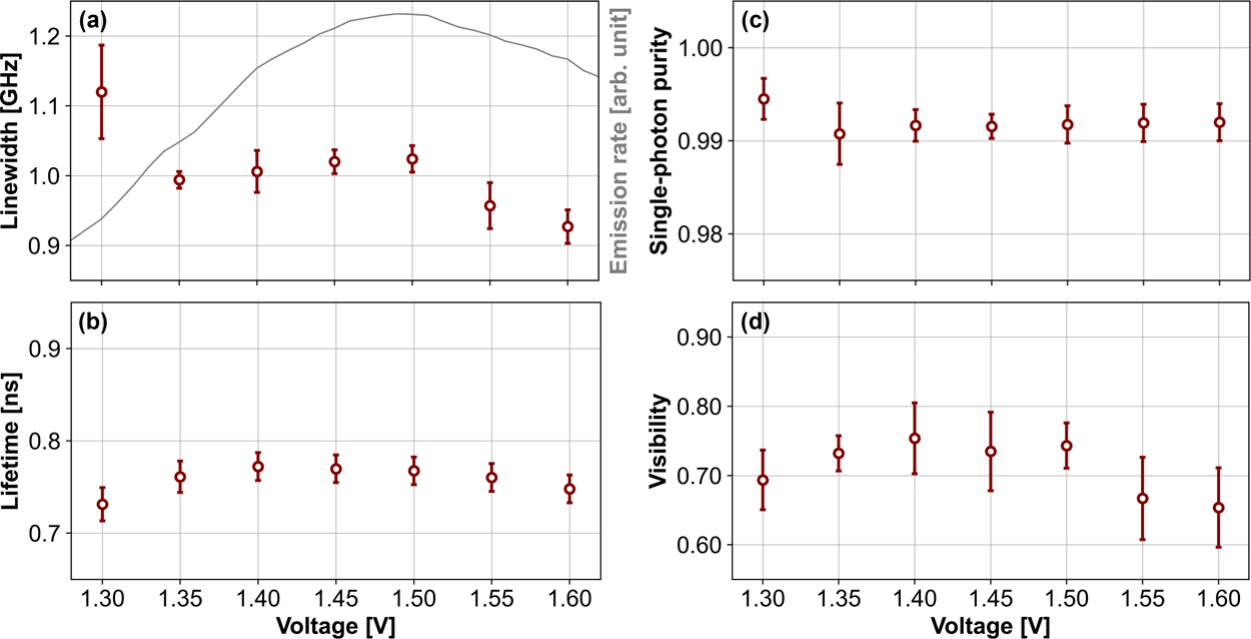
Emission line width (a), the emission lifetime (b), the single-photon purity (c), and the visibility (d) of the X^–^ emission line of eCBG2 under quasi-resonant p-shell excitation, measured at 7 different bias voltages. The corresponding voltage-dependent emission rates are additionally plotted (gray solid line) in (a).

Furthermore, extending from the quasi-resonant measurements, the emission line width, the emission lifetime, the single-photon purity, and the visibility were observed under seven different bias voltages, as illustrated in Figure 7. The voltage range was selected around 1.45 V, where the X^–^ emission was the brightest under quasi-resonant excitation (gray solid line). It is noteworthy that the emission rate dropped more drastically when the voltage decreased, and only about 20% of the maximum intensity was observed at 1.3 V, leading to a larger uncertainty in the line width measurement at that voltage. However, the highest single-photon purity and shortest emission lifetime were observed at the same voltage (1.3 V), while the best visibility was observed at 1.4 V instead. When considering the estimated uncertainties of the data points (from the standard deviations of multiple repeated measurements), no strong voltage dependency on these properties was observed. For the best performance, the voltage where the emission rate is maximized (in this case, around 1.45 V) is recommended.

## Conclusions

In this report, we proposed and implemented designs for high-performance electrically controlled QD-CBG single-photon sources. We explored two different ridge-based eCBG designs based on the trade-off between electrical and optical performance. The sensitivity of the electrical path length and the ridge width to the performance prompted careful optimizations between numerical and experimental results. A direct-ridge configuration featured better electrical connection, requiring a slightly narrower minimum ridge width for an electrically functioning device while optically performing poorer, especially for the polarization in parallel to the ridge axis. On the other hand, a mazy-ridge configuration featured a slightly longer electrical path length, requiring a wider minimum ridge width to function, but yielded superior optical enhancement. The deterministically fabricated mazy-ridge QD-eCBG achieved a PEE of 30.4(3.4)%, in quantitative agreement with numerical simulations which promise substantial improvement with PEE > 50% when using reoptimized parameters based on the present experiment findings. The electrical operation of the QD-eCBG yields precise charge control and a quantum-confined Stark effect, providing the robust capability to fine-tune the emission wavelength up to 0.7 meV and selectively enhance emission lines from specific QD states. The single-photon emission characteristics show an impressive single-photon purity of 99.2(2)% and raw visibility of 0.75(5) under quasi-resonant p-shell excitation. The results underscore the high potential of our advanced quantum device concept, which can be used in many applications of photonic quantum information technology requiring not only excellent optical and quantum optical properties but also tight electrical control of the integrated quantum emitters.

## Experimental Methods

### Optical Setups

The μPL measurements were performed on the sample cooled to 4 K in a closed-cycle cryostat using a Ti:sapphire laser operating in pulsed picosecond mode (80 MHz) at tunable wavelengths (800–865 nm). The emitted photons were collected with a cold aspheric objective lens (NA = 0.81). With a beam splitter, which separates excitation and collection paths, the collected beam was directed through a 900 nm long-pass filter to a monochromator with a 1500-line-per-mm grating and detected using a CCD camera. For time-resolved measurements, two different correlation setups, equipped with SNSPDs, were used: (1) HBT setup, where the spectrally filtered photons were sent through a 1:1 single-mode fiber beam splitter and detected with two SNSPDs, and (2) HOM setup, where the spectrally filtered photons were sent through a polarization-maintaining beam splitter in combination with polarization-control paths (with λ/2- and λ/4-plates) and a ∼4 ns time delay on one arm, before being sent back into a 1:1 beam splitter for two-photon interference effect and detected with two SNSPDs. In these correlation setups, the time correlation between two detected photons in each detector was recorded.

## Supporting Information Available

Summary of relevant CBG works based on AlGaAs material system and simulated PEEs of the optimized eCBG devices with spatial mismatches (PDF)
