# Supplementary material for: Bright Electrically Contacted Circular Bragg Grating Resonators with Deterministically Integrated Quantum Dots
Source: ACS Nano. 2024 Nov 5;18(46):31834–45. doi: 10.1021/acsnano.4c07820 (PMC11580368; doi:10.1021/acsnano.4c07820)
Supplement: Supplementary file 1 — nn4c07820_si_001.pdf [file nn4c07820_si_001.pdf]

Supporting Information: Bright electrically contacted circular Bragg grating resonators with deterministically integrated quantum dots

Setthanat Wijitpatima,† Normen Auler,‡ Priyabrata Mudi,† Timon Funk,† Avijit Barua,† Binamra Shrestha,‡ Johannes Schall,† Imad Limame,† Sven Rodt,† Dirk Reuter,‡ and Stephan Reitzenstein*,†

†*Institute of Solid State Physics, Technische Universität Berlin, Hardenbergstraße 36, Berlin 10623, Germany*

‡*Department of Physics, Paderborn University, Warburger Str. 100, 33098 Paderborn, Germany*

E-mail: stephan.reitzenstein@physik.tu-berlin.de

S1 Relevant works

Relevant works and their circular Bragg grating (CBG) cavity designs based on the AlGaAs material system are summarized in Table S1.

Table S1: Relevant references and their circular Bragg grating (CBG) cavity designs based on AlGaAs material system, with reported simulated (sim.) and experimental (exp.) photon extraction efficiency (PEE) and Purcell factor (F_P). In each work, the first lens with a different numerical aperture (NA) was used. The dash ("-") symbols indicate that the specific information is not reported in the corresponding work. The grey (yellow) shaded references focus on CBG designs without (with) electrical controls. Linewidth (LW) and electrical tuning range shown were calculated in µeV for comparison. The number in the parenthesis in the reference list represents the published year (20xx).

Design		Exp.	NA	PEE [%]		F_P		LW [µeV]	Tuning range [meV]	Reference
Cavity	Backside			Sim.	Exp.	Sim.	Exp.			
p-CBG [1]	Air	Yes	0.42	53	**10**	12	**4**	100	-	Davanço (11)[1]<br>Ates (12)[2]
p-CBG	Air	Yes	0.4	50	**48**	11	**3**	-	-	Sapienza (15)[3]
CBG	SiO2/Au	No	0.65	96	**-**	19	**-**	-	-	Yao (18)[4]
CBG	SiO2/Au	Yes	0.65	90	**85**	2	**3.5**	-	-	Liu (19)[5]
CBG	SiO2/Au	Yes	0.65	90	**79.5**	20	**11.3**	-	-	Wang (19)[6]
Ridged CBG	Air	No	-	-	**-**	-	**-**	-	-	Ji (21)[7]
Ridged CBG	SiO2/Au	No	0.65	70	**-**	20	**-**	-	-	Barbiero (22)[8]
Ridged CBG	Air	Yes	-	-	**-**	-	**15.35**	-	-	Singh (22)[9]
Ridged CBG	SiO2/Au	No	0.64	83	**-**	18	**-**	-	-	Buchinger (23)[10]
CBG [2]	SiO2/Au	No	0.8	91.1	**-**	27.8	**-**	-	-	Rickert (23)[11]
Ridged CBG	DBR	Yes	0.81	32	**31**	-	**1.4**	4	700	**This work**

[1]p-CBG stands for partially etched CBG.
[2]Rickert et al. (2023) purposed a device with a deposited layer of transparent conductive material on CBG to function as electrical gate.

S2 Effect of QD-eCBG spatial mismatch

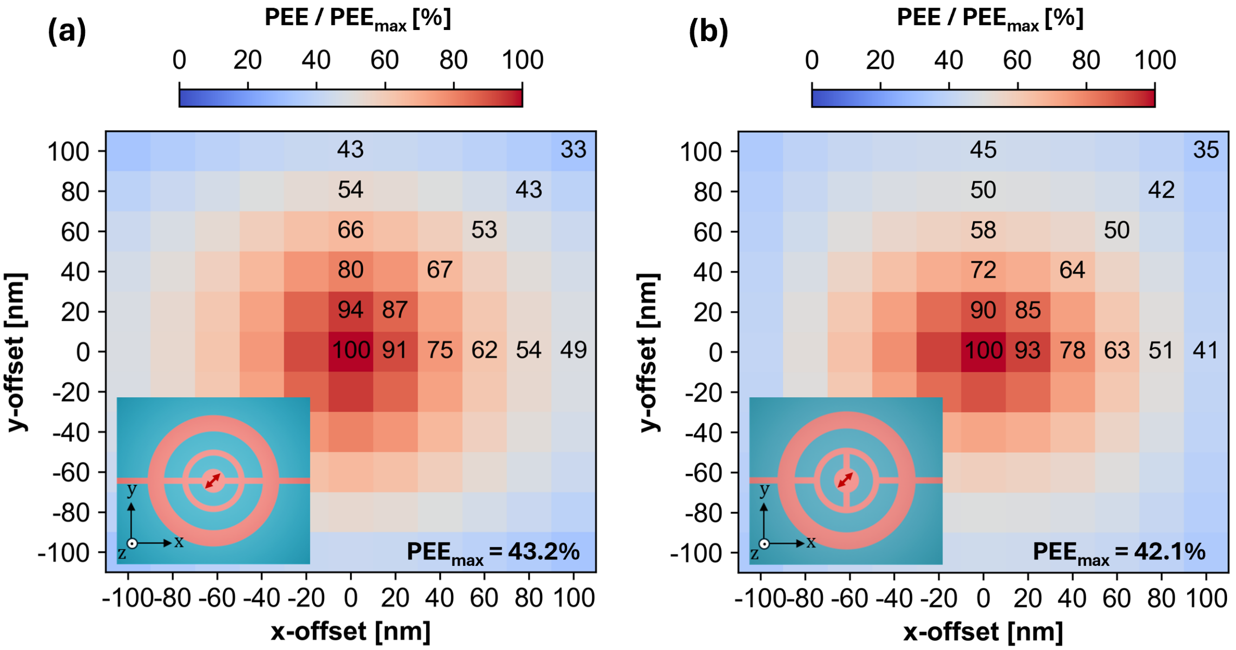

Figure S1: Simulated PEEs of eCBG devices based on the optimized parameters in Table 1 with spatial mismatches between QD and eCBG in direct-ridge (a) and mazy-ridge (b) configurations. The FEM simulation employed a linear dipole source oriented 45º relative to the x-axis located at the positions with x- and y-offsets from the center of the eCBG structures.

By performing additional FEM simulations based on the optimized device parameters shown in Table 1, with a linear dipole source oriented 45º relative to the x-axis located at the positions with x- and y-offsets from the center of the eCBG, the results shown in Figure S1 indicate that, in both direct-ridge and mazy-ridge cases, the PEEs drop by at most 50% from their peak values (occurring when QD is perfectly spatially aligned) within the displacement of 80 nm. Within the offset of 20 nm, which can be achieved using deterministic marker-based EBL,[12] the PEE of above 90% of the maximum can be secured.

References

(1) Davanço, M.; Rakher, M. T.; Schuh, D.; Badolato, A.; Srinivasan, K. A Circular Dielectric Grating for Vertical Extraction of Single Quantum Dot Emission. *Applied Physics Letters* **2011**, *99*, 041102.

(2) Ates, S.; Sapienza, L.; Davanco, M.; Badolato, A.; Srinivasan, K. Bright Single-Photon Emission from a Quantum Dot in a Circular Bragg Grating Microcavity. *IEEE Journal of Selected Topics in Quantum Electronics* **2012**, *18*, 1711–1721.

(3) Sapienza, L.; Davanço, M.; Badolato, A.; Srinivasan, K. Nanoscale Optical Positioning of Single Quantum Dots for Bright and Pure Single-Photon Emission. *Nature Communications* **2015**, *6*, 7833.

(4) Yao, B.; Su, R.; Wei, Y.; Liu, Z.; Zhao, T.; Liu, J. Design for Hybrid Circular Bragg Gratings for a Highly Efficient Quantum-Dot Single-Photon Source. *Journal of the Korean Physical Society* **2018**, *73*, 1502–1505.

(5) Liu, J.; Su, R.; Wei, Y.; Yao, B.; Silva, S. F. C. D.; Yu, Y.; Iles-Smith, J.; Srinivasan, K.; Rastelli, A.; Li, J.; Wang, X. A Solid-State Source of Strongly Entangled Photon Pairs with High Brightness and Indistinguishability. *Nature Nanotechnology* **2019**, *14*, 586–593.

(6) Wang, H.; Hu, H.; Chung, T.-H.; Qin, J.; Yang, X.; Li, J.-P.; Liu, R.-Z.; Zhong, H.-S.; He, Y.-M.; Ding, X.; Deng, Y.-H.; Dai, Q.; Huo, Y.-H.; Höfling, S.; Lu, C.-Y.; Pan, J.-W. On-Demand Semiconductor Source of Entangled Photons Which Simultaneously Has High Fidelity, Efficiency, and Indistinguishability. *Physical Review Letters* **2019**, *122*, 113602.

(7) Ji, S.; Tajiri, T.; Kiyama, H.; Oiwa, A.; Iwamoto, S. Design of Bull's-Eye Optical Cavity toward Efficient Quantum Media Conversion Using Gate-Defined Quantum Dot. *Japanese Journal of Applied Physics* **2021**, *60*, 102003.

(8) Barbiero, A.; Huwer, J.; Skiba-Szymanska, J.; Müller, T.; Stevenson, R. M.; Shields, A. J. Design Study for an Efficient Semiconductor Quantum Light Source Operating in the Telecom C-Band Based on an Electrically-Driven Circular Bragg Grating. *Optics Express* **2022**, *30*, 10919.

(9) Singh, H.; Farfurnik, D.; Luo, Z.; Bracker, A. S.; Carter, S. G.; Waks, E. Optical Transparency Induced by a Largely Purcell Enhanced Quantum Dot in a Polarization-Degenerate Cavity. *Nano Letters* **2022**, *22*, 7959–7964.

(10) Buchinger, Q.; Betzold, S.; Höfling, S.; Huber-Loyola, T. Optical Properties of Circular Bragg Gratings with Labyrinth Geometry to Enable Electrical Contacts. *Applied Physics Letters* **2023**, *122*, 111110.

(11) Rickert, L.; Betz, F.; Plock, M.; Burger, S.; Heindel, T. High-Performance Designs for Fiber-Pigtailed Quantum-Light Sources Based on Quantum Dots in Electrically-Controlled Circular Bragg Gratings. *Optics Express* **2023**, *31*, 14750.

(12) Rickert, L.; Vajner, D. A.; von Helversen, M.; Schall, J.; Rodt, S.; Reitzenstein, S.; Liu, H.; Li, S.; Ni, H.; Niu, Z.; Heindel, T. High Purcell-Enhancement in Quantum-Dot Hybrid Circular Bragg Grating Cavities for GHz-Clockrate Generation of Indistinguishable Photons. 2024; `https://arxiv.org/abs/2408.02543`.
